# Supplementary material for: The mammalian rod synaptic ribbon is essential for Cav channel facilitation and ultrafast synaptic vesicle fusion
Source: eLife. 2021 Oct 7;10:e63844. doi: 10.7554/eLife.63844 (PMC8594941; doi:10.7554/eLife.63844)
Supplement: Supplementary file 1. [file elife-63844-supp1.docx]

**Supplementary File 1**. Whole-cell patch-clamp recording parameters

|  | Lockin-Amplifier outputs | | |  | |
| --- | --- | --- | --- | --- | --- |
| *Ribeye* wt  0.5mM EGTA  (21 cells) | Cm  (Farads) | Gm  (Siemens) | Gs  (Siemens) | Ra  (MOhm) | Leak at  -70mV  (Ampere) |
| *Mean* | 974f | 67.7p | 28.3n | 31.3M | -4.83p |
| *SE of mean* | 39.8f | 11.5p | 2.03n | 3.74M | 928f |
| *Minimum* | 460f | 7.30p | 14.0n | 14.8M | -17.0p |
| *Median* | 987f | 45.0p | 28.0n | 21.5M | -3.20p |
| *Maximum* | 1.35p | 220p | 54.0n | 88.0M | 0.00 |
| *Ribeye* (-/-)  0.5mM EGTA  (17 cells) | Cm  (Farads) | Gm  (Siemens) | Gs  (Siemens) | Ra  (MOhm) | Leak at  -70mV  (Ampere) |
| *Mean* | 1.05p | 88.4p | 32.0n | 27.1M | -6.26p |
| *SE of mean* | 62.8f | 20.5p | 3.82n | 2.91M | 1.61p |
| *Minimum* | 679f | 15.1p | 11.2n | 11.7M | -20.0p |
| *Median* | 948f | 49.3p | 30.8n | 22.3M | -2.30p |
| *Maximum* | 1.67p | 306p | 71.7n | 57.7M | 0.00 |
| *Ribeye* wt  10mM EGTA  (21 cells) | Cm  (Farads) | Gm  (Siemens) | Gs  (Siemens) | Ra  (MOhm) | Leak at  -70mV  (Ampere) |
| *Mean* | 1.01p | 58.3p | 23.2n | 29.0M | -3.35p |
| *SE of mean* | 20.4f | 8.65p | 1.23n | 3.64M | 839f |
| *Minimum* | 897f | 14.0p | 12.8n | 12.8M | -12.0p |
| *Median* | 995f | 54.1p | 23.5n | 20.9M | -3.00p |
| *Maximum* | 1.28p | 137p | 31.1n | 68.4M | 1.50p |
| *Ribeye* (-/-)  10mM EGTA  (10 cells) | Cm  (Farads) | Gm  (Siemens) | Gs  (Siemens) | Ra  (MOhm) | Leak at  -70mV  (Ampere) |
| *Mean* | 974f | 39.5p | 21.9n | 37.4M | -3.57p |
| *SE of mean* | 162f | 34.3p | 8.16n | 13.8M | 3.79p |
| *Minimum* | 665f | 10.4p | 9.99n | 18.9M | -12.0p |
| *Median* | 968f | 25.9p | 21.4n | 42.0M | -2.00p |
| *Maximum* | 1.21p | 111p | 37.1n | 51.7M | -1.00p |
