# Supplementary material for: The mammalian rod synaptic ribbon is essential for Cav channel facilitation and ultrafast synaptic vesicle fusion
Source: eLife. 2021 Oct 7;10:e63844. doi: 10.7554/eLife.63844 (PMC8594941; doi:10.7554/eLife.63844)
Supplement: Supplementary file 2. [file elife-63844-supp2.docx]

| Supplementary File 2. Activation kinetics for Ca^2+^-currents measured from wild type and *Ribeye-*ko rods filled with 0.5 or 10 mM EGTA.  Comparison of different intracellular concentrations of EGTA (within each genotype) | | | | | | | | | | | | | | |
| --- | --- | --- | --- | --- | --- | --- | --- | --- | --- | --- | --- | --- | --- | --- |
| V_step_ | | -40mV | | -30mV | | -20mV | | -10mV | | | | 0mV | | |
|  | | peak-I_Ca_ (pA) | | peak-I_Ca_ (pA) | tau (µs) | peak-I_Ca_ (pA) | tau (µs) | peak-I_Ca_ (pA) | | tau (µs) | | peak-I_Ca_ (pA) | tau (µs) | |
| wt, 10 EGTA  9 cells | | -0.9 ± 0.3 | | -6.2 ± 0.6 | 1090 ± 151 | -12.1 ± 0.7 | 489 ± 42 | -14.1 ± 0.7 | | 274 ± 36 | | -13.0±0.7 | 184 ± 27 | |
| wt 0.5 EGTA  8 cells | | -0.9 ± 0.1 | | -6.1 ± 0.3 | 545 ± 30  **p: 0.006** | -12.2 ± 0.4 | 309 ± 29  **p: 0.003** | -14.1 ± 0.6 | | 202 ± 22  p: 0.09 | | -13.5±0.4  p: 0.52 | 155 ± 34  p: 0.51 | |
| ko, 10 EGTA  5 cells | | -0.5 ± 0.1 | | -3.7 ± 0.7 | 671 ± 65 | -7.7 ± 0.8 | 363 ± 27 | -9.3 ± 0.7 | | 255± 36 | | -9.8 ± 0.7 | 261 ± 35 | |
| ko, 0.5 EGTA  7 cells | | -1.0 ± 0.1  **p: 0.006** | | -6.0 ± 0.5  **p: 0.028** | 649 ± 67  **p: 0.82** | -9.4 ± 0.7  p: 0.13 | 254 ± 42  **p: 0.05** | -11.3 ± 0.9  p: 0.10 | | 194± 17  p: 0.18 | | -9.7 ± 0.7 | 140 ± 18  **p: 0.02** | |
| Comparison of wild type versus *Ribeye-*ko | | | | | | | | | | | | | | |
| V_step_ | -40mV | | -30mV | | | -20mV | | | -10mV | | 0mV | | | |
|  | peak-I_Ca_ (pA) | | peak-I_Ca_ (pA) | | tau (µs) | peak-I_Ca_ (pA) | tau (µs) | | peak-I_Ca_ (pA) | tau (µs) | peak-I_Ca_ (pA) | | | tau (µs) |
| 10 EGTA wt | -0.9 ± 0.3 | | -6.2 ± 0.6 | | 1090 ± 151 | -12.1 ± 0.7 | 489 ± 42 | | -14.1 ± 0.7 | 274 ± 36 | -13.0 ± 0.7 | | | 184± 27 |
| 10 EGTA  ko | -0.5 ± 0.1  p: 0.34 | | -3.7 ± 0.7  **p:0.023** | | 671 ± 65  **p: 0.03** | -7.7 ± 0.8  **p:0.002** | 363 ± 27  **p: 0.03** | | -9.3 ± 0.7  **p: 0.0008** | 255± 36  p: 0.7 | -9.8 ± 0.7  **p: 0.03** | | | 261± 35  p: 0.12 |
| 0.5 EGTA  wt | -0.9 ± 0.1 | | -6.1 ± 0.3 | | 545 ± 30 | -12.2 ± 0.4 | 309 ± 29 | | -14.1 ± 0.6 | 202 ± 22 | -13.5 ± 0.4 | | | 155± 34 |
| 0.5 EGTA  ko | -1.0 ± 0.1  p: 0.47 | | -6.0 ± 0.5 | | 649 ± 67  p: 0.19 | -9.4 ± 0.7  **p: 0.005** | 254 ± 42  p: 0.3 | | -11.3±0.9  **p: 0.02** | 194± 17  p: 0.78 | -9.7 ± 0.7  **p: 0.0002** | | | 140± 18  p: 0.7 |

**Note:** Average peak-I_Ca_ and activation τ's were made by fitting current responses with a single exponential decay function (see Materials and methods). The voltage-step protocol started at V_rest_: −70 mV, stepped to the test voltage (V_step_) for 10 ms, and a 3 s rest period was given before the next test pulse. The protocol started with a V_step_ to +30 mV, advanced in 10 mV decrements, and ended with a step to −80 mV. The liquid junction potentials (*E*_lj_) was *not* subtracted from the voltage values presented above. The *E*_lj_ created with the intracellular solutions used to make 0.5 and 10 mM EGTA are estimated to shift the membrane voltages negatively by 10 and 9 mV, respectively (see Materials and methods).
