# Supplementary material for: The mammalian rod synaptic ribbon is essential for Cav channel facilitation and ultrafast synaptic vesicle fusion
Source: eLife. 2021 Oct 7;10:e63844. doi: 10.7554/eLife.63844 (PMC8594941; doi:10.7554/eLife.63844)
Supplement: Supplementary file 3. — Comparison of different intracellular concentrations of EGTA (within each genotype). [file elife-63844-supp3.docx]

**Supplementary File 3.** Peak-I_Ca_−Voltage relationship for wild type and *Ribeye-*ko rods filled with 0.5 or 10 mM EGTA.

Comparison of different intracellular concentrations of EGTA (within each genotype)

| genotype, EGTA conc. | Modified Boltzmann-IV fits | | | | | Boltzmann fits | | |
| --- | --- | --- | --- | --- | --- | --- | --- | --- |
|  | I_Ca_ (pA)  @-10mV | V_1/2_  (mV) | dx  (mV-e^−1^) | V_rev_  (mV) | G_max_  (pA-mV^−1^) | *peak-I_Ca_*  *(pA)* | *V_0.5_*  *(mV)* | *dx*  *(mV-e^−1^)* |
| wt, 10 EGTA  9 cells | -14.1 ± 0.7 | -24.0 ± 1.3 | -6.2 ± 0.6 | 44.6 ± 2.6 | 0.30 ± 0.02 | -14.5 ± 0.3 | -28.7 ± 0.4 | 4.8 ± 0.4 |
| wt, 0.5 EGTA  8 cells | -14.1 ± 0.6 | -23.2 ± 0.9 | -6.2 ± 0.6 | 46.4 ± 2.6 | 0.29 ± 0.02 | -14.4 ± 0.2 | -28.6 ± 0.2 | 4.7 ± 0.2 |
| ko, 10 EGTA  5 cells | -9.3 ± 0.7 | -22.3 ± 1.9 | -6.9 ± 0.8 | 43.2 ± 2.9 | 0.22 ± 0.02 | -10.0 ± 0.2 | -27.3 ± 0.44 | 5.5 ± 0.4 |
| ko, 0.5 EGTA  7 cells | -11.3 ± 0.9  p: 0.13 | -25.3 ± 1.1  p: 0.17 | -6.3 ± 0.7 | 43.9 ± 3.1 | 0.22 ± 0.02 | -11.5 ± 0.82  p: 0.15 | -30.3 ± 1.1  p: 0.053 | 5.2 ± 1.1  p: 0.83 |
| Comparison of wild type versus *Ribeye-*ko | | | | |  |  |  |  |
| wt 10 EGTA | -14.1 ± 0.7 | -24.0 ± 1.3 | -6.2 ± 0.6 | 44.6 ± 2.6 | 0.30 ± 0.02 | -14.5 ± 0.3 | -28.7 ± 0.4 | 4.8 ± 0.4 |
| ko 10 EGTA | -9.3 ± 0.7  **p: 0.0008** | -22.3 ± 1.9  p: 0.46 | -6.9 ± 0.8 | 43.2 ± 2.9 | 0.22 ± 0.02  **p: 0.024** | *-*10.0 ± 0.02  **p: 0.0001** | -27.3 ± 0.44  **p: 0.033** | 5.5 ± 0.4  **p: 0.28** |
| wt 0.5 EGTA | -14.1 ± 0.6 | -23.2 ± 0.9 | -6.2 ± 0.6 | 46.4 ± 2.6 | 0.29 ± 0.02 | -14.4 ± 0.2 | -28.6 ± 0.2 | 4.7 ± 0.2 |
| ko 0.5 EGTA | -11.3 ± 0.9  **p: 0.020** | -25.3 ± 1.1  p: 0.17 | -6.3 ± 0.7 | 43.9 ± 3.1 | 0.22 ± 0.02  **p: 0.029** | -11.5 ± 0.82  **p: 0.003** | -30.3 ± 1.1  p: 0.18 | 5.2 ± 1.1  p: 0.64 |

Notes: Average peak-I_Ca_ versus V_step_ curves were fit with a Boltzmann equation to derive the values stated above (see Materials and methods for equations). The voltage step protocol is described in the legend to Supplementary File 2. Liquid junction potentials were not subtracted from the voltage values presented above (i.e., V_1/2_, V_rev_ and V_0.5_); see Supplementary File 2 for details.
