# Supplementary material for: The mammalian rod synaptic ribbon is essential for Cav channel facilitation and ultrafast synaptic vesicle fusion
Source: eLife. 2021 Oct 7;10:e63844. doi: 10.7554/eLife.63844 (PMC8594941; doi:10.7554/eLife.63844)
Supplement: Supplementary file 4. [file elife-63844-supp4.docx]

**Supplementary File 4**. Comparison of Ca^2+^-activated Cl^-^-tail currents measured from wild type and *Ribeye-*ko rods filled with 0.5 mM EGTA.

| genotype | I_Cl(Ca)_ @ −10 mV (pA) | Modified Boltzmann-IV fits | | | | Boltzmann fits | | |
| --- | --- | --- | --- | --- | --- | --- | --- | --- |
|  |  | V_1/2_  (mV) | dx  (mV-e^−1^) | V_rev_  (mV) | G_max_  (pA-mV^−1^) | span  (pA) | V_0.5_  (mV) | dx  (mV-e^−1^) |
| wt 0.5 EGTA  n: 6 | −68 ± 13 | −20.4 ± 0.5 | −5.55 ± 0.95 | 35.8 ± 3.8 | 1.41 ± 0.44 | 67 ± 14 | −23.9 ± 0.8 | 3.20 ± 0.19 |
| ko 0.5 EGTA  n: 5 | −27 ± 5  **p: 0.036** | −22.8 ± 0.6  **p: 0.016** | −5.28 ± 0.25  p: 0.79 | 34.1 ± 3.0  p: 0.73 | 0.70 ± 0.13  *p: 0.17* | 27 ± 5  **p: 0.042** | −27.4 ± 0.8  **p: 0.013** | 3.52 ± 0.24  p: 0.33 |

Notes: Ca^2+^-activated Cl^-^-tail currents were generated with the same voltage step protocol used to measure peak-I_Ca_ amplitude (see the legend to Supplementary File 3), and Boltzmann fits were made as described in Materials and methods. Tail-current amplitudes were measured 3 ms after repolarizing the cell to −70 mV. Liquid junction potentials were *not* subtracted from the voltage values presented above (i.e., V_1/2_, V_rev_ and V_0.5_); see Supplementary File 2 for details.
